# MR extracellular volume mapping and non-contrast T1ρ mapping allow early detection of myocardial fibrosis in diabetic monkeys

**DOI:** 10.1007/s00330-018-5950-9

**Published:** 2019-01-14

**Authors:** Yu Zhang, Wen Zeng, Wei Chen, Yushu Chen, Tong Zhu, Jiayu Sun, Zhigang Liang, Wei Cheng, Lei Wang, Bing Wu, Li Gong, Victor A. Ferrari, Jie Zheng, Fabao Gao

**Affiliations:** 1 Department of Radiology, West China Hospital, Sichuan University, 37 Guoxue Alley, Chengdu, 610041 Sichuan China; 2 Sichuan Primed Shines Bio-tech Co., Ltd., Chengdu, China; 3 The First Affiliated Hospital of Kunming Medical University, Kunming, China; 4 Cardiovascular Division, Perelman School of Medicine, Hospital of the University of Pennsylvania, Philadelphia, USA; 5 Mallinckrodt Institute of Radiology, Washington University in St Louis, 4525 Scott Ave, Room 3114, St. Louis, MO 63110 USA

**Keywords:** Fibrosis, Type 2 diabetes mellitus, Rhesus monkey, Diastole, Extracellular matrix

## Abstract

**Objective:**

To detect diffuse myocardial fibrosis in different severity levels of left ventricular diastolic dysfunction (DD) in spontaneous type 2 diabetes mellitus (T2DM) rhesus monkeys.

**Methods:**

Eighteen spontaneous T2DM and nine healthy monkeys were studied. Echocardiography was performed for diastolic function classification. Cardiac magnetic resonance (CMR) imaging was performed to obtain extracellular volume fraction (ECV) maps and T1ρ maps at two different spin-locking frequencies. ECV values, T1ρ values, and myocardial fibrosis index (mFI) values which are based on the dispersion of T1ρ, were calculated. Global peak diastolic longitudinal strain rates (GSrL) were also obtained.

**Results:**

Echocardiography results showed mild DD in nine T2DM monkeys and moderate DD in the other nine. The global ECV values were significantly different among healthy animals as compared with animals with mild DD or moderate DD, and the ECV values of animals with moderate DD were significantly higher as compared with those of mild DD. The mFI values increased progressively from healthy animals to those with mild DD and then to those with moderate DD. Diastolic function indicators (e.g., early diastolic mitral annulus velocity, GSrL) correlated well with ECV and mFI.

**Conclusions:**

Monkeys with T2DM exhibit increased ECV, T1ρ, and mFI values, which may be indicative of the expansion of extracellular volume and the deposition of excessive collagen. T1ρ mapping may have the potential to be used for diffuse myocardial fibrosis assessment.

**Key Points:**

• *Monkeys with T2DM exhibit increased ECV, T1ρ, and mFI values, which may be indicative of the expansion of extracellular volume and the deposition of excessive collagen*.

• *The relationship between diastolic dysfunction and diffuse myocardial fibrosis may be demonstrated by imaging markers*.

• *Non-contrast T1ρ mapping may have the potential to be used for diffuse myocardial assessment*.

**Electronic supplementary material:**

The online version of this article (10.1007/s00330-018-5950-9) contains supplementary material, which is available to authorized users.

## Introduction

Diabetes leads to abnormalities in cardiac relaxation that predominantly results in heart failure with preserved ejection fraction (HFpEF) [1]. The number of comorbidities in patients with HFpEF is higher, and the clinical outcomes are worse as compared with those found in patients with heart failure with reduced ejection fraction (HFrEF) [2–4]. Left ventricular diastolic dysfunction (DD) has been recognized as the pathophysiological cornerstone of HFpEF. Diffuse myocardial fibrosis and extracellular matrix remodeling may be the major causes of DD [5, 6]. However, the early detection of diffuse fibrosis at different severity levels of DD in diabetes with HFpEF has not been fully investigated.

Cardiac magnetic resonance (CMR) T1 mapping has been used for the assessment of diffuse myocardial fibrosis. However, post-contrast T1 mapping is affected by the agent dose, the measurement timing, and the renal clearance rate. Although native T1 has recently emerged as a non-contrast imaging technique for the assessment of myocardial fibrosis [7, 8], the corresponding change in native T1 to different quantities of fibrosis appears to be limited. In contrast, extracellular volume fraction (ECV) is not affected by many external factors, thus permitting more accurate comparisons for diffuse myocardial fibrosis quantification [9]. Meanwhile, an endogenous contrast technique known as *native T1ρ mapping* has been used for myocardial fibrosis detection [10, 11]. In one study, a positive correlation between T1ρ and ECV was observed in 20 patients with dilated cardiomyopathy [12]. In addition, to improve the sensitivity of T1ρ mapping for myocardial fibrosis assessment, T1ρ dispersion contrast has been adopted [13, 14]. There is a real need for non-contrast techniques for patients with renal dysfunction or for whom contrast is otherwise contraindicated [15].

Rhesus monkeys with spontaneous type 2 diabetes mellitus (T2DM) demonstrated manifestations that are highly similar to those of human beings [16]. This animal model provides a unique opportunity to investigate the mechanisms of complications that arise from T2DM.

In this initial study, rhesus monkeys with T2DM and varying degrees of DD were used for diffuse myocardial fibrosis detection. We used ECV as an imaging marker to determine diffuse myocardial fibrosis content. In addition, the potential of non-contrast T1ρ mapping for the assessment of diffuse myocardial fibrosis was also explored.

## Methods

### Animals

Eighteen rhesus monkeys with spontaneous T2DM (11 to 19 years old, with 1- to 5-year duration of T2DM) and nine healthy control (HC) rhesus monkeys matched for gender, age, and weight were studied. The criteria used for rhesus monkey selection were guided by our previous work [17] and required a fasting plasma glucose (FPG) level of 5.5 mmol/L or more and a glycated hemoglobin (HbA1c) level of 4.5% or more [16, 18]. None of the monkeys had a history of medical treatment. All rhesus monkeys used in this study were provided by Sichuan Primed Bio-Tech Co., Ltd. (Sichuan, China). The monkeys were maintained in accordance with the requirements of the National Institutes of Health Guide for the Care and Use of Laboratory Animals and the Association for Assessment and Accreditation of Laboratory Animal Care. All experimental protocols were reviewed and approved by the Experimental Animal Ethics Committee of West China Hospital, Sichuan University, Chengdu 610041(Institutional approved number 2015065A) and by the Institutional Animal Care and Use Committee of Sichuan Primed Group Co., Ltd.

The general condition and related blood biochemical indicators of all monkeys are shown in Table 1. Information about animal preparation before echocardiographic and CMR examination can be found in the supplements.

**Table 1:** Basic characteristics

	Healthy control animals (*N* = 9), group 1	Monkeys with type 2 diabetes and mild diastolic dysfunction (*N* = 9), group 2	Monkeys with type 2 diabetes and moderate diastolic dysfunction (*N* = 9), group 3	*p* value*
Male sex	9	8 (88.89%)	9	
Age (years)	13.33 ± 1.11	14.55 ± 2.60	14.11 ± 1.88	0.496
Body weight (kg)	10.15 ± 0.84	10.94 ± 1.42	10.83 ± 1.53	0.513
Diabetes duration (years)	0	1–5	1–5	
FPG (mmol/L)	4.24 (4.05–4.48)	6.20 (5.53–6.32)^‡^	6.43 (6.08–6.58)^‡^	0.000
HbA1c (%)	4.35 (4.30–4.45)	5.10 (4.80–5.30)^‡^	4.90 (4.75–5.10)^‡^	0.000
FRA (μmol/L)	179.40 ± 8.56	194.57 ± 14.01	199.40 ± 15.82	0.075
TC (μmol/L)	3.05 ± 0.60	3.61 ± 0.67	3.12 ± 0.59	0.172
TG (mmol/L)	0.38 ± 0.14	0.51 ± 0.25	0.51 ± 0.31	0.464
HDL-c (mmol/L)	1.77 ± 0.58	2.30 ± 0.67	1.84 ± 0.88	0.263
LDL-c (mmol/L)	1.39 ± 0.49	1.48 ± 0.28	1.21 ± 0.44	0.358
BUN (mmol/L)	5.13 ± 0.96	5.18 ± 1.33	4.06 ± 0.53	0.180
Scr (μmol/L)	103.75 ± 20.79	103.00 ± 12.05	102.75 ± 19.97	0.996
ALT (IU/L)	56.30 ± 25.73	47.52 ± 23.05	46.80 ± 18.38	0.757
AST (IU/L)	29.98 ± 7.16	23.52 ± 5.77	24.90 ± 8.02	0.292
GGT (IU/L)	67.80 ± 15.93	74.71 ± 17.27	85.40 ± 23.21	0.356
Mean SBP (mm HG)	125.50 ± 10.61	130.35 ± 23.65	132.76 ± 15.45	0.576
Mean DBP (mm HG)	68.75 ± 9.90	63.48 ± 8.68	67.42 ± 5.13	0.657

Values are given as mean ± standard deviation, *N* (%), or median (Q1–Q3)

*p* value was the results of one-way analysis of variance (for normally distributed date) or Kruskal-Wallis *H* test (for non-normally distributed date). *ALT*, glutamic-pyruvic transaminase; *AST*, glutamic-oxaloacetic transaminase; *BUN*, blood urea nitrogen; *DBP*, diastolic blood pressure; *FPG*, fasting plasma glucose; *FRA*, fructosamine; *GGT*, gamma-glutamyl transpeptidase; *HbA1c*, glycated hemoglobin; *HDL-c*, serum high-density lipoprotein; *LDL-c*, serum low-density lipoprotein; *SBP*, systolic blood pressure; *Scr*, serum creatinine; *TC*, serum total cholesterol; *TG*, serum triglyceride; *Q*, quartile

*Compared between three groups

^‡^*p* < 0.05 compared with group 1 (according to the results of Fisher’s least significant difference test and Student-Newman-Keuls test or Dunn-Bonferroni test for post hoc analysis)

### Echocardiography

Transthoracic echocardiography was performed using a standard protocol [19] with the GE Vivid S5 hand-carried Doppler system (GE Medical Systems, Israel Ltd.). Various diastolic function indicators were all measured, including the mitral early diastolic peak (E-wave) and the late peak (A-wave) velocities, the E/A ratio, the early diastolic mitral annulus peak velocity (E’), the late diastolic mitral annulus peak velocity (A’), the E/E’ ratio, and the E’/A’ ratio. Data were averaged across three cardiac cycles.

In accordance with the classification of DD in our previous study and in humans [20, 21], monkeys were divided into three DD severity groups for data analysis. Monkeys with normal diastolic function (i.e., E/A > 1, E’/A’ > 1, E’ > 7, and E/E’ < 11) were assigned to group 1. Monkeys that show E/A < 1, E’/A’ > or < 1, E’ > 7, and E/E’ < 11 were classified as having mild DD and assigned to group 2, and monkeys that show E/A > 1, E’/A’ < 1, E’ < 7, and E/E’ > 11 were classified as having moderate DD and assigned to group 3.

### Cardiac magnetic resonance

CMR imaging was performed using a 3.0 Tesla magnetic resonance imaging system (Magnetom Trio; Siemens Medical Systems) with a 32-channel cardiac surface coil (Siemens).

A CMR cine sequence was performed to scan 10 to 14 short-axis slices via a steady-state free precession with retrospective electrocardiogram triggering. Next, the T1ρ images were obtained using an electrocardiogram-gated T1ρ-prepared gradient echo sequence in three short-axis slices at the base, middle, and apex of the left ventricle. A modified look-locker inversion recovery (MOLLI) sequence with motion correction was then applied to obtain pre-contrast T1 maps with an acquisition scheme of 5(3)3 [22, 23]. Ten minutes after contrast injection (0.15 mmol/kg of Magnevist; Bayer Health Care Pharmaceuticals), post-contrast MOLLI T1 mapping was acquired with a scheme of 4(1)3(1) [22, 23] in the same three short-axis slices that were used for the T1ρ scans. Late gadolinium enhancement images were obtained using an electrocardiogram-gated breath-hold inversion recovery Turbo FLASH at 15 min after contrast injection (Table 2).

**Table 2:** Cardiac magnetic resonance imaging parameters

Sequences	Cine	T1ρ mapping	T1 mapping	Late gadolinium enhancement
Field of view read (mm)	160	160	160	160
Field of view phase (mm)	125	140	140	135
Slice thickness (mm)	5.0	5.0	5.0	5.0
Interp. pixel size (mm^2^)	0.83 × 0.83	1.25 × 1.25	0.83 × 0.83	0.63 × 0.63
Acq. pixel size (mm^2^)	1.25 × 1.49	1.67 × 1.67	1.67 × 1.67	0.63 × 0.63
Matrix size (read *x* phase)	128 × 84	96 × 84	96 × 84	256 × 162
Readout time (ms)	52	40	138	105
Repetition time (ms)	26.5	134	362	335
TR (ms)/TE (ms)	3.22/1.41	3.6/1.5	2.6/1.12	4.6 / 2.1
Averages	1	1	1	2
Number of TI/mode	N/A	N/A	Pre-contrast: MOLLI-5-3-3 post-contrast: MOLLI-4-1-3-1-2	N/A
K-space lines/RR	8	11	53	23
GRAPPA factor	2	N/A	2	2
Partial Fourier	6/8	N/A	N/A	N/A
Bandwidth (Hz/Px)	449	801	1078	287
Spin-locking frequency	N/A	510 Hz or 0 Hz	N/A	N/A
Time of spin-locking (ms)	N/A	10, 30, 50	N/A	N/A
Scan time (s)	5.4	19	7	10
Flip angle	12^o^	15^o^	35^o^	20^o^

All CMR images were prospectively analyzed. A blood sample was taken from all monkeys immediately before each CMR study for hematocrit measurements. Cine and ECV mapping were analyzed offline with cmr^42^© software (Circle Cardiovascular Imaging Inc.). The following parameters were obtained: left ventricular end-systolic volume (LVESV), left ventricular end-diastolic volume (LVEDV), stroke volume (SV), left ventricular ejection fraction (LVEF), left ventricular (LV) mass, and average heart rate (a-HR). Based on the cine sequence, we also performed strain analysis using cmr^42^©-based feature tracking. Both the global peak systolic longitudinal strain (GSL) and the global peak diastolic longitudinal strain rate (GSrL) were obtained. Based on the pre-contrast and post-contrast T1 maps, which were automatically generated with a prototype inline process function from Siemens, ECV maps were generated by cmr^42^©. If there was motion between the pre- and post-T1 series, the pre- and post-MOLLI were registered using a cmr42© intensity-based registration method then ECV maps were calculated. The myocardial fibrosis index (mFI) was calculated as follows:

1 $$ \mathrm{mFI}\left({\omega}_1\right)=T{1}_{\rho}\left({\omega}_1\right)-T{1}_{\rho }(0), $$

where the spin-locking frequency (SLF) is *ω*_1_ = *γB*_1_ and *γ* is the gyromagnetic ratio. The *T*1_*ρ*_(0) value is relatively constant for myocardial tissue with different quantities of fibrosis. Although the mFI is calculated in millisecond, it was used as an index that had been calculated in arbitrary units for the purposes of this study. All of the image processing and analyses were performed with a custom-written software (ImPro_MR_Analysis_Suite), which was created in MATLAB (MathWorks). Segmental [24] ECV values, mFI values, and T1ρ values were all acquired. Whole-heart average values were obtained, with the exclusion of artifact segments. Since mFI represents the subtraction of two T1ρ images, the artifacts exclusion of T1ρ and mFI is based on the original T1ρ-weighted images. Signal intensity values of more than the mean plus 5 multiplied by the standard deviation of the signal intensity in remote normal myocardium were used to determine regional fibrosis.

### Histology

One diabetic monkey was given deep anesthesia (pentobarbital sodium, P3761; Sigma-Aldrich) to euthanasia. Histologic slices from the anterior interventricular septum were excised. After dehydration and embedding, the slices were subjected to the Masson staining protocol and analyzed under an optical microscope (BX43F; Olympus).

### Statistics

Statistical analysis was performed using SPSS version 22 (IBM). All data were checked for normality using the Shapiro-Wilk test and presented as the mean ± standard deviation and mean (95% confidence interval (CI)) or as a median (Q1–Q3), as appropriate. Normally distributed data sets were analyzed with one-way analysis of variance, and Fisher’s least significant difference test and Student-Newman-Keuls test were used in post hoc analysis. The Kruskal-Wallis *H* test was used to analyze non-normal distribution; the Dunn-Bonferroni test was used in post hoc analysis. Bivariate correlations were performed using either the Pearson or Spearman method, as appropriate. Significance was assumed at *p* < 0.05.

## Results

### Echocardiography

There were nine T2DM monkeys with mild DD in group 2 and nine T2DM monkeys with moderate DD in group 3. The nine HC monkeys in group 1 demonstrated normal DD (Supplemental Table 1).

### Cardiac magnetic resonance

**Fig. 1:**
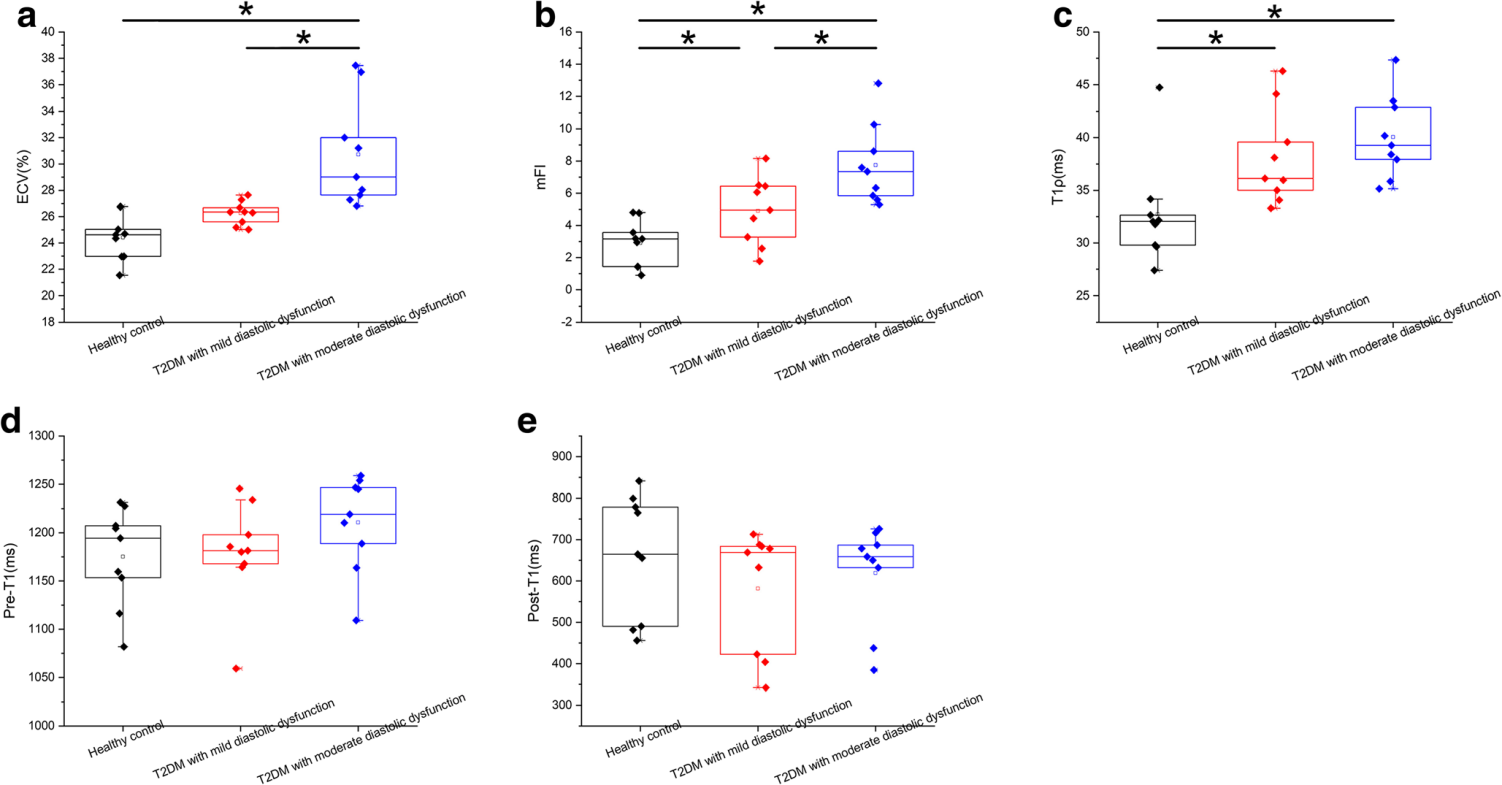
Results for animals with different degrees of diastolic dysfunction as compared with healthy controls. Differences in extracellular volume fraction (**a**), myocardial fibrosis index (**b**), T1ρ relaxation time (**c**), pre-contrast myocardial T1 (**d**), and post-contrast myocardial T1 (**e**) are compared between healthy control animals, monkeys with type 2 diabetes mellitus and mild diastolic dysfunction, and monkeys with type 2 diabetes mellitus and moderate diastolic dysfunction. Values in panels a–e are shown in box plots. One-way analysis of variance was used for comparing the differences among the three groups (this method was used for normally distributed data, including T1ρ, mFI, and pre-T1). For post hoc analysis, Fisher’s least significant difference test and the Student-Newman-Keuls test were used to compare the differences between every two groups. The Kruskal-Wallis H test was used to compare the differences among the three groups (this method was used for non-normally distributed data, including ECV and post-T1). The Dunn-Bonferroni test was used in post hoc analysis to compare the differences between every two groups. *p < 0.05

**Fig. 2:**
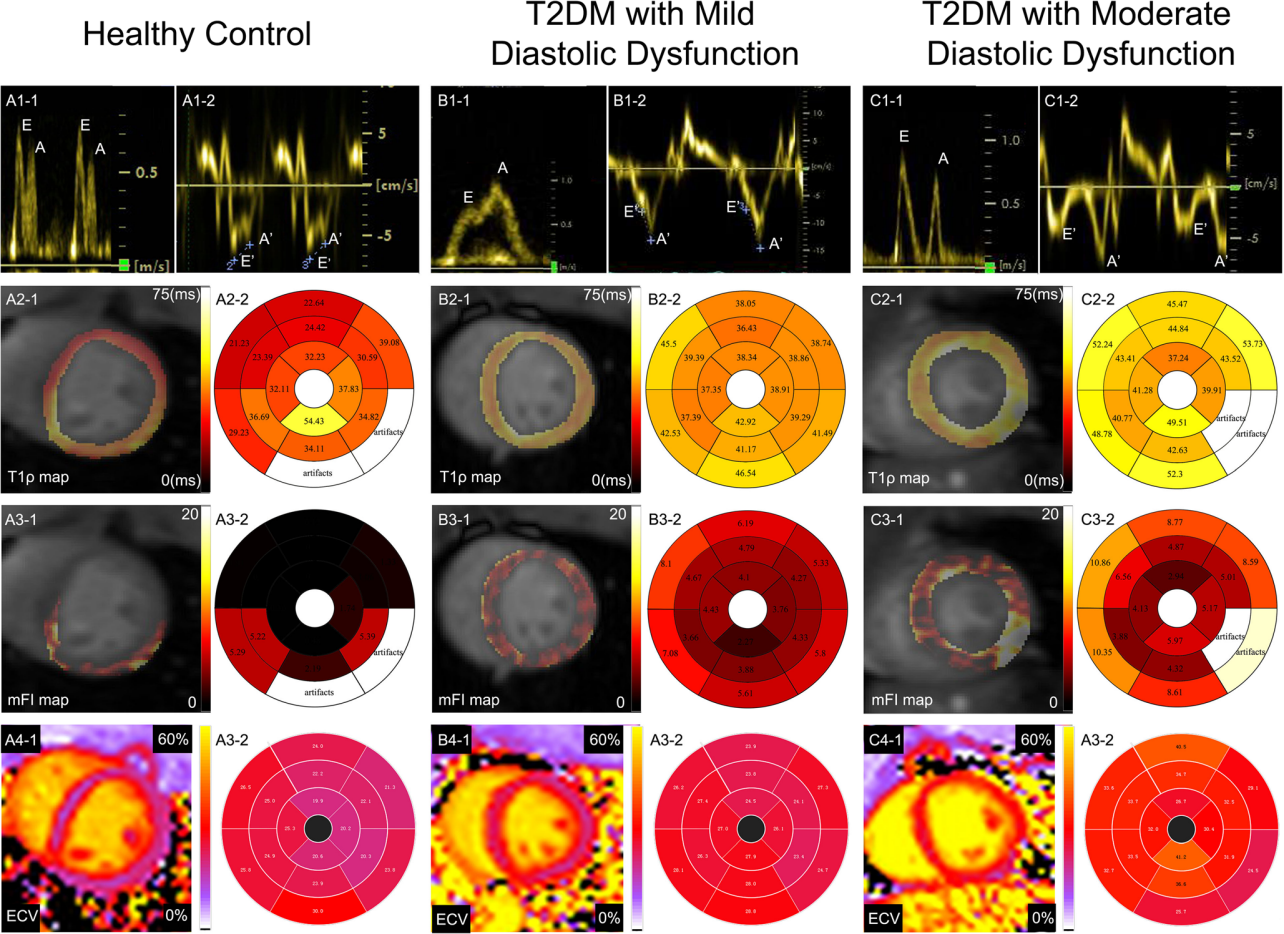
Imaging examples of animals with different degrees of diastolic dysfunction and healthy control animals. The first row displays the echocardiographic mitral valve inflow (**A1**, **B1**, **C1**–**1**) and the septal mitral valve annular velocities (**A1**, **B1**, **C1**–**2**). The second row shows the T1ρ maps (**A2**, **B2**, **C2**–**1**) and the corresponding bull’s-eye plots (**A2**, **B2**, **C2**–**2**). The third row shows mFI maps (**A3**, **B3**, **C3**–**1**) and the corresponding bull’s-eye plots (**A3**, **B3**, **C3**–**2**). The fourth row shows the extracellular volume fraction maps (**A4**, **B4**, **C4**–**1**) and the corresponding bull’s-eye plots (**A4**, **B4**, **C4**–**2**). Differences in fibrosis content among the three groups of monkeys were clearly indicated by three fibrosis imaging markers with different image contrasts

The baseline CMR results summarized in Table 3 show that there were no significant differences in LVESV, LVEDV, SV, LVEF, LV mass, and a-HR among the three groups. Despite the non-significant findings related to the basic cardiac function of the three groups, the global myocardial ECV (%) was significantly different among the three groups (24.61 (22.96–25.88) vs. 26.34 (25.39–26.97) vs. 29.02 (27.46–34.48); *p* < 0.000), and the ECV values of group 3 were significantly higher as compared with those of group 2 (*p* = 0.027). However, there was no significant difference in ECV values between group 1 and group 2 (*p* = 0.307) (Figs. 1 and 2). There was no evidence of regional late contrast enhancement in any of the monkeys. On the other hand, T1ρ relaxation time was significantly higher in groups 2 and 3 as compared with group 1; however, there was no significant difference between group 2 and group 3. The mFI increased progressively from healthy animals to those with mild DD and then to those with moderate DD (2.90 (1.82–4.00) vs. 4.91 (3.30–6.51) vs. 7.74 (5.84–9.65); *p* < 0.000). The differences between group 1 and group 2 (*p* = 0.049) and between group 2 and group 3 (*p* = 0.007) were all significant. GSrL values in group 3 were significantly lower than those of group 1 (*p* = 0.013). The results of the post hoc analysis are shown in Supplemental Table 2.

**Table 3:** Cardiac magnetic resonance characteristics

	Healthy control animals (*N* = 9), group 1	Monkeys with Type 2 diabetes and mild diastolic dysfunction (*N* = 9), group 2	Monkeys with type 2 diabetes and moderate diastolic dysfunction (*N* = 9), group 3	*p* value*
LVEDV (mL)	16.82 (13.29–23.57)	18.86 (17.43–20.09)	22.70 (16.64–27.86)	0.225
LVEDV/BW (mL/kg)	1.73 (1.43–2.24)	1.64 (1.48–1.95)	1.95 (1.73–2.44)	0.275
LVESV (mL)	8.24 (4.15–12.34)	8.79 (5.83–11.75)	9.09 (6.15–12.04)	0.917
LVESV/ BW (mL/kg)	0.79 (0.44–1.14)	0.81 (0.52–1.11)	0.85 (0.56–1.15)	0.945
Stroke volume (mL)	9.21 (7.31–12.34)	9.36 (7.81–11.64)	13.87 (10.03–18.34)	0.068
LVEF (%)	57.06 (44.71–69.42)	53.39 (42.88–66.91)	61.14 (55.85–66.43)	0.526
LV mass S (g)	19.78 (16.77–22.80)	21.97 (19.31–24.57)	23.35 (17.36–29.34)	0.375
LV mass D (g)	16.24 (14.41–18.07)	20.21 (16.91–23.53)	21.41 (14.33–28.50)	0.115
Heart rate (bpm)	99.01 (77.79–120.24)	97.02 (82.58–111.48)	103.04 (89.60–116.49)	0.774
Global myocardial T1ρ (ms)	32.71 (28.92–36.50)	38.06 (34.60–41.53)^‡^	40.06 (37.05–43.06)^‡^	0.006
Global mFI	2.90 (1.82–4.00)	4.91 (3.30–6.51)^‡^	7.74 (5.84–9.65)^‡†^	0.000
Global pre-contrast myocardial T1 time (ms)	1175.16 (1135.67–1214.66)	1179.53 (1138.72–1220.36)	1199.47 (1172.44–1248.73)	0.580
Global post-contrast myocardial T1 time (ms)	664.79 (486.01–788.91)	669.14 (413.33–685.59)	658.69 (534.92–701.63)	0.461
Global ECV (%)	24.61 (22.96–25.88)	26.34 (25.39–26.97)	29.02 (27.46–34.48)^‡†^	0.000
HCT (%)	43.00 (41.10–45.80)	42.30 (40.35–44.85)	43.70 (38.05–45.00)	0.505
Global myocardial T2 (ms)	35.83 (31.12–40.54)	42.22 (36.56–47.88)	39.50 (33.42–45.58)	0.187
GSrL (1/s)	1.28 (1.05–1.53)	0.74 (0.66–1.21)	0.72 (0.58–0.95)^‡^	0.015
GSL (%)	− 12.99 (− 13.89 to − 10.40)	− 10.34 (− 10.82 to − 5.92)	−9.43 (− 11.06 to – 4.58)	0.094

Values are given as mean (95% CI) or median (Q1–Q3)

*p* value was the results of one-way analysis of variance (for normally distributed date) or Kruskal-Wallis *H* test (for non-normally distributed date). *BW*, body weight; *CI*, confidence interval; *ECV*, extracellular volume fraction; *GSL*, global peak systolic longitudinal strain; *GSrL*, global peak diastolic longitudinal strain rate; *HCT*, hematocrit; *LV*, left ventricular; *LVEDV*, left ventricular end-diastolic volume; *LVEF*, left ventricular ejection fraction; *LVESV*, left ventricular end-systolic volume; *mFI*, myocardial fibrosis index; *Q*, quartile

*Compared between three groups

^‡^*p* < 0.05 compared with group 1 (according to the results of Fisher’s least significant difference test and Student-Newman-Keuls test or Dunn-Bonferroni test for post hoc analysis)

^†^*p* < 0.05 compared with group 2 (according to the results of Fisher’s least significant difference test and Student-Newman-Keuls test or Dunn-Bonferroni test for post hoc analysis)

### Correlation between the extracellular volume fraction and other CMR-derived diffuse myocardial fibrosis markers

**Fig. 3:**
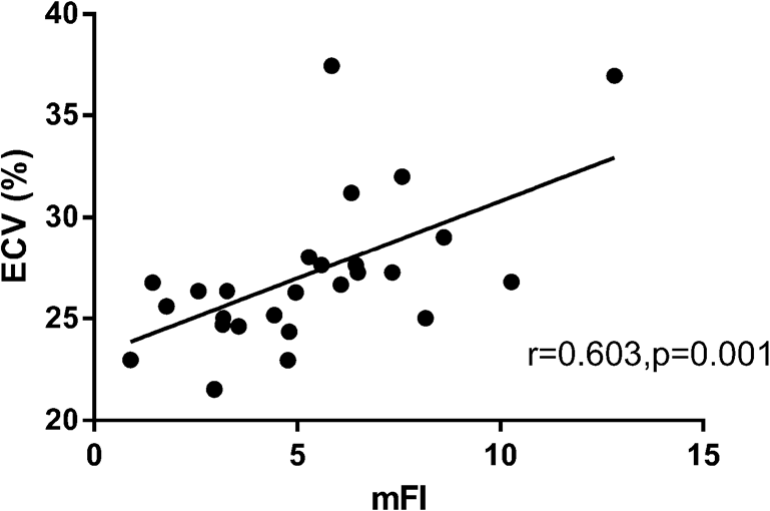
Correlation between extracellular volume fraction and the myocardial fibrosis index. A positive correlation was observed between the T1 mapping-derived extracellular volume fraction and the T1ρ mapping-derived myocardial fibrosis index

There was a moderate positive correlation between ECV and mFI (*r* = 0.603) (Fig. 3). A positive correlation between ECV and T1ρ relaxation time was also found (*r* = 0.582).

### Correlation between imaging markers of diffuse myocardial fibrosis and cardiac function indicators

**Fig. 4:**
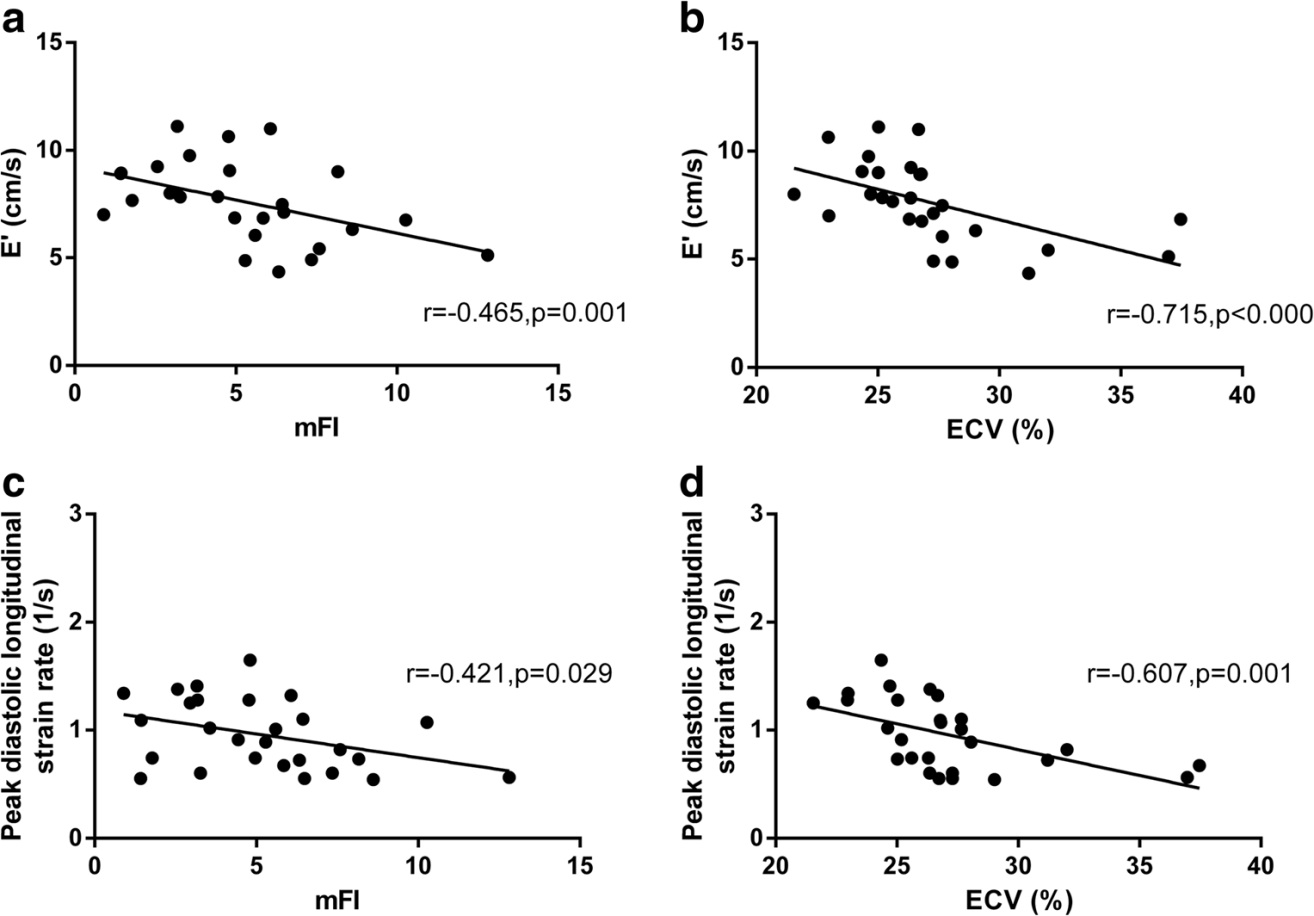
Correlation between imaging markers of diffuse myocardial fibrosis and diastolic function. **a** A negative correlation was observed between E’ and the myocardial fibrosis index. **b** A negative correlation was observed between E’ and the extracellular volume fraction. **c** A negative correlation was found between the peak diastolic longitudinal strain rate and the myocardial fibrosis index. **d** A negative correlation was found between the peak diastolic longitudinal strain rate and the extracellular volume fraction

GSrL had a negative correlation with mFI (*r* = − 0.421) and ECV (*r* = − 0.607). GSL was found to be correlated with ECV (*r* = − 0.434). E’ was correlated with mFI (*r* = − 0.465), T1ρ (*r* = − 0.549), and ECV (*r* = − 0.715) (Table 4; Fig. 4).

**Table 4:** Correlation between cardiac magnetic resonance derived diffuse myocardial fibrosis markers and myocardial function

Variable	mFI correlation coefficient	*p* value	T1 ρ correlation coefficient	*p* value	ECV correlation coefficient	*p* value
GSrL (1/s)	− 0.421	0.029	− 0.377	0.053	− 0.607	0.001
GSL (%)	0.243	0.222	0.297	0.132	0.434	0.024
E (cm/s)	0.167	0.404	− 0.111	0.580	0.060	0.768
E/A	− 0.213	0.285	− 0.207	0.300	− 0.099	0.623
E’ (cm/s)	− 0.465	0.014	− 0.549	0.003	− 0.715	0.000
E’/A’	− 0.470	0.013	− 0.570	0.002	− 0.660	0.000
E/E’	0.352	0.072	0.303	0.125	0.528	0.005

*p* value was the results of Pearson method (for normally distributed date) or Spearman method (for non-normally distributed date). *A*, transmitral late diastolic filling velocity; *A’*, late diastolic mitral annulus velocity; *E*, transmitral early diastolic filling velocity; *E’*, early diastolic mitral annulus velocity; *GSL*, global peak systolic longitudinal strain; *GSrL*, global peak diastolic longitudinal strain rate

### Histology

**Fig. 5:**
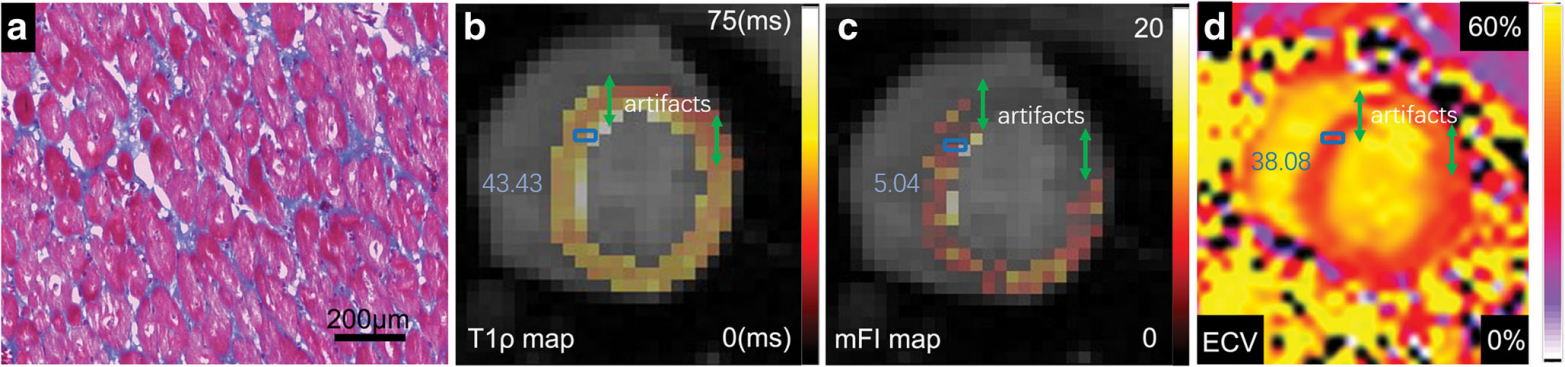
Masson staining and corresponding cardiac magnetic resonance images from one monkey with moderate diastolic dysfunction. Masson staining is shown under high-power magnification (× 100). (**a**) Collagen fibers were stained with aniline blue, and cardiomyocytes were stained red. The blue rectangles in the T1ρ map (**b**), the myocardial fibrosis index map (**c**), and the extracellular volume fraction map (**d**) indicate the approximate areas form which the histopathological slides were taken. The values in each map indicate the mean T1ρ, myocardial fibrosis index, and extracellular volume fraction values measured in the blue rectangles

Perivascular and interstitial fibrosis were confirmed in this T2DM monkey with moderate DD with the use of Masson staining (Fig. 5). Collagen fibers were stained with aniline blue, and cardiomyocytes were stained red. The corresponding CMR ECV map and mFI map are also shown in Fig. 5. The average values of ECV and mFI in the approximate areas from which the histopathological slides were taken were found to be elevated (ECV = 37.46%; mFI = 5.85).

## Discussion

Diffuse myocardial fibrosis in monkeys with T2DM and DD was detected with the use of CMR. Monkeys with T2DM exhibited increased ECV, T1ρ, and mFI values, which may be indicative of the expansion of extracellular volume and the deposition of excessive collagen. A moderate correlation between mFI and ECV (an established surrogate marker of diffuse myocardial fibrosis) was observed. A relatively strong association between imaging markers of diffuse myocardial fibrosis (i.e., mFI and ECV) and diastolic function indicators was also observed.

In this study, rhesus monkeys with spontaneous T2DM were used. This non-human primate (NHP) model shares metabolic and pathological features with humans [18]. Li Gong et al [16] reported the classification and diagnosis of T2DM in the rhesus monkey subspecies *Macaca mulatta lasiotis*, which was used in the present study. Like humans, the NHPs have an increased likelihood of developing obesity and T2DM with increasing age [25]. Monkeys in this study lived in a highly controlled and stable environment, and their metabolic histories were well documented. The use of this model is critical for pharmaceutical companies to develop and test drugs. Before this model can be used appropriately, the cardiovascular complications that develop in these animals need to be well characterized. Non-invasive imaging methods allow for the longitudinal monitoring of therapeutic effects; however, the ground truth confirmation of disease must be obtained during the last stages of these monkeys’ lives.

The methods used in our study to measure diastolic function were based on well-established echocardiographic techniques. The echocardiography findings presented in this study only demonstrated DD in monkeys with T2DM. These results concurred with the findings of the studies by Can [21] and Haihua [26] in diabetic monkeys. The classification of DD in our study is based on our coworkers’ previous study as well as data from humans [20, 21]. Sedated monkeys were tested under stable conditions to alleviate motion problems. A new and sensitive indicator—the GSrL, which was based on CMR cine sequences [27]—was also used in diastolic function measurement. The GSrL was observed to be decreased in patients with T2DM as compared with lean or obese controls [28, 29]. In our study, the GSrL in monkeys with T2DM was also lower.

DD in T2DM with HFpEF is manifested as impaired relaxation and decreased diastolic chamber compliance [30]. Collagen deposition around intramural cardiac vessels and between myofibers as well as expanded extracellular space is thought to be important contributors to DD in the presence of T2DM [31]. In our study, both ECV and mFI were correlated with E’ and GSrL. Su and colleagues [32] studied patients with HFrEF and patients with HFpEF, and those authors found that ECV correlated well with peak filling rate, which is a diastolic functional index assessed by cine, only in patients with HFpEF. This again suggests that diffuse fibrosis is a key factor in the pathophysiology of DD.

As has been demonstrated in previous studies in humans [33, 34], ECV values in monkeys with T2DM were elevated. The use of T1ρ contrast to assess myocardial fibrosis has been reported in several swine studies in which T1ρ values as much as doubled in the myocardial scar tissue as compared with the normal tissue [10, 13]. In our study, non-contrast T1ρ values were found to increase in monkeys with T2DM as compared with HCs, but there was no significant difference between animals with mild DD and animals with moderate DD. However, mFI values were significantly different between animals with mild DD and animals with moderate DD as well as between HCs and animals with mild DD. A recent animal study [14] provided additional evidence that mFI has a greater sensitivity for detecting diffuse types of myocardial fibrosis as compared with T1ρ in dogs with myocardial infarction.

Despite the performance of a few studies in ex vivo tissue, in vivo animals, and human patients [11, 13, 35], the precise mechanisms by which T1ρ detects myocardial fibrosis remain unknown. The chemical exchange of γB_1_ on a time scale or in an intermediate exchange regimen is likely to play an important role in the modulation of T1ρ signals [36]. In accordance with the chemical exchange theory [37], an increased concentration of water protons bound to macromolecules (e.g., collagen) leads to increases in mFI. The magnitude of the increase is modulated by the chemical shifts and exchange rates of the macromolecules. The higher sensitivity of mFI to the changes in collagen content may be due to the cancelation of intrinsic T2, thereby resulting in an amplified effect of the chemical exchange during the relaxation times, when water protons to exchange are locked without dephasing [14]. It was noted previously that mFI represents the subtraction of two T1ρ images. In theory, mFI cannot have a value of zero due to the presence of collagens. However, as a result of the noise in the measurement, the values of mFI in normal myocardial tissue can be close to zero.

Although a good correlation between mFI and ECV was found, there were still differences. The precise mechanisms that account for the different behaviors of ECV and mFI and that allow them to differentiate HCs from animals with mild DD remain unknown. One reason may be that increased ECV reflects the expansion of extracellular volume whereas increased mFI reflects the deposition of excessive collagen. In a recent study by Shiro and colleagues [38], ECV was strongly correlated (*r* = 0.86) with the histological extracellular space component but only modestly correlated (*r* = 0.66) with the histological collagen volume fraction in patients with dilated cardiomyopathy. Future studies to systematically validate the mFI method with the use of histologically defined collagen volume are warranted, and they should include a sufficient sample size. Meanwhile, the relationship between collagen deposition and extracellular volume expansion is still in need of further investigation.

### Limitations

Due to the use of this rare, naturally occurring, chronic, NHP disease model of monkeys with T2DM as well as the longitudinal long-term study consideration, only one monkey with T2DM and DD was sacrificed for histopathology to show diffuse myocardial fibrosis. The ECV measurements in this study had not previously been validated in a monkey model. Given the close agreement of ECV among various species [39, 40], it is reasonable to assume that the ECV measurement in a monkey is still valid, although direct rigorous validation remains to be performed by histopathology. While we did monitor blood pressures immediately before and after CMR study, we did not have pressure data during the CMR study to normalize CMR results. This limitation will be resolved with the addition of MRI-compatible physiological monitoring at our institute [41]. Finally, although there are statistically significant differences in ECV and mFI between the different monkey groups, there is also an overlapping of values. Further studies with larger simple sizes are needed for vigorous validation beyond this initial investigation.

## Conclusions

In this initial study, monkeys with T2DM exhibited increased ECV, T1ρ, and mFI values, which may be indicative of the expansion of extracellular volume and the deposition of excessive collagen. The relationship between diastolic dysfunction and diffuse myocardial fibrosis was demonstrated by imaging markers. T1ρ mapping may have the potential to be used for the assessment of diffuse myocardial fibrosis.

## Electronic supplementary material

ESM 1 (DOCX 32 kb)

## Abbreviations

A: Transmitral late diastolic filling velocity
A’: Late diastolic mitral annulus velocity
a-HR: Average heart rate
AGE-RAGE: Advanced glycation end products/receptors for glycation end products
CMR: Cardiac magnetic resonance
DD: Diastolic dysfunction
E: Transmitral early diastolic filling velocity
E’: Early diastolic mitral annulus velocity
ECV: Extracellular volume fraction
FPG: Fasting plasma glucose
FOV: Field of view
GSL: Global peak systolic longitudinal strain
GSrL: Global peak diastolic longitudinal strain rate
HbA1c: Glycated hemoglobin
HC: Healthy control
HFpEF: Heart failure with preserved ejection fraction
LVEDV: Left ventricular end-diastolic volume
LVEF: Left ventricular ejection fraction
LVESV: Left ventricular end-systolic volume
mFI: Myocardial fibrosis index
MOLLI: Modified look-locker inversion
NHP: Non-human primate
SLF: Spin-locking frequency
SV: Stroke volume
T2DM: Type 2 diabetes mellitus
TE: Echo time
TR: Repetition time
TSL: Time of spin-locking
