# Supplementary material for: MR extracellular volume mapping and non-contrast T1ρ mapping allow early detection of myocardial fibrosis in diabetic monkeys
Source: Eur Radiol. 2019 Jan 14;29(6):3006–16. doi: 10.1007/s00330-018-5950-9 (PMC6510861; doi:10.1007/s00330-018-5950-9)
Supplement: Supplementary file 1 — (DOCX 32 kb) [file 330_2018_5950_MOESM1_ESM.docx]

**SUPPLEMENTAL MATERIAL**

**Ethics approval and consent to participate**

All rhesus monkeys used in this study were provided by Sichuan Primed Bio-Tech Co., Ltd. (Sichuan, China). The monkeys were maintained in accordance with the requirements of the National Institutes of Health Guide for the Care and Use of Laboratory Animals and the Association for Assessment and Accreditation of Laboratory Animal Care. All experimental protocols were reviewed and approved by the Experimental Animal Ethics Committee of West China Hospital, Sichuan University, Chengdu 610041, and by the Institutional Animal Care and Use Committee of Sichuan Primed Group Co., Ltd.

**METHODS**

**Study Protocol**

There was a 4-week acclimation period before the initial imaging study during which gravimetric and metabolic profiles of all studied monkeys were acquired. Fasting plasma glucose (FPG) levels were determined semi-monthly for 4 weeks. Glycated hemoglobin (HbA1c), total cholesterol, triglyceride, low-density lipoprotein cholesterol, high-density lipoprotein cholesterol, and other metabolic profiles were determined at the end of the acclimation period. Body weight was measured after the last blood collection. Blood pressure was measured before echocardiography examination. Echocardiography was performed on all monkeys within 48 hours after the final blood collection. Cardiac magnetic resonance was performed sequentially on all monkeys. One diabetic monkey was specifically sacrificed for histopathology.

**Animal Maintenance**

All monkeys were singly housed in a climate-controlled room at 19°C to 26°C, with a relative humidity of 50% ± 20%. The rate of ventilation was 10 air changes per hour, and the lighting cycle was 12 hours of day and 12 hours of night. All monkeys were fed approximately 300 g/day of standard monkey fodder containing 17% protein, 68% carbohydrates, and 15% fat. A daily allotment of apples or vegetables was also provided, and ad libitum tap water was provided for drinking.

**Animal preparation before each examination**

**Echocardiographic**

All monkeys were fasted overnight before echocardiography examination. They were sedated with ketamine hydrochloride (10 mg/ml; Bioniche Teoranta, Inverin Co) at a dosage of 10 mg/kg given intramuscularly. The skin of each monkey was shaved bilaterally from the cervical area to the navel and then rinsed copiously with water so that acoustic coupling could be obtained with the use of ultrasound gel. The preparation process was performed as in our previous study[1].

**Cardiac Magnetic Resonance**

The right antecubital vein passage was established in each animal before scanning. The animals were anesthetized with the use of ketamine hydrochloride (10 mg/kg given intramuscularly), and this was followed by tracheal intubation with artificial ventilation to control respiration (tidal volume, 150 to 200 mL/min; respiratory rate, 15 to 20 breaths/min; isoflurane dosage, 0.2 to 0.3 mL/kg; United States Pharmacopeia grade 100%; RWD Life Science). Each monkey was placed in a comfortable supine position in the scanner. Data were acquired during end-inspiratory breath holding of 10 to 15 seconds.

**Cardiac Magnetic Resonance imaging analysis**

All CMR images were prospectively analyzed. A blood sample was taken from all monkeys immediately before each CMR study for hematocrit measurements. Cine sequence and ECV mapping were analyzed offline with the use of cmr^42©^ software (Circle Cardiovascular Imaging Inc.). The short-axis stack was analyzed semi-automanually, with contouring of the endocardial and epicardial borders from base to apex at the end-diastole phase and the end-systole phase, respectively. The following parameters were obtained: left ventricular end-systolic volume (LVESV), left ventricular end-diastolic volume (LVEDV), stroke volume (SV), left ventricular ejection fraction (LVEF), left ventricular (LV) mass and average heart rate (a-HR). Based on the cine sequences, we also performed strain analysis using cmr^42©^-based feature tracking. Both the global peak systolic longitudinal strain (GSL) and the global peak diastolic longitudinal strain rate (GSrL) were obtained. For ECV map analysis, precontrast and postcontrast T1 maps of three short axes were automatically generated with a prototype inline process function from Siemens. In cmr^42©^, endocardial and epicardial views of each slice’s maps were semi-automanually contoured, with care taken to avoid blood pool. Then, a region of interest was placed in the blood pool. With the use of hematocrit values, ECV maps were also generated. The mFI can be calculated as follows:

$mFI\left( \omega_{1} \right)={{T1}_{\rho}\left( \omega_{1} \right)-T1}_{\rho}\left( 0 \right)$, (1)

where the spin-locking frequency (SLF) is ω_1_ = γB_1_ and γ is the gyromagnetic ratio. The ${T1}_{\rho}(0)$ value is relatively constant for myocardial tissue with different quantities of fibrosis. Although the mFI is calculated in ms, it was used as an index that had been calculated in arbitrary units for the purposes of this study. All of the image processing and analyses were performed with the use of a custom-written software (ImPro_MR_Analysis_Suite), which was created in MATLAB (MathWorks). In brief, the ${T1}_{\rho}$-weighted signals with three different TSL were fitted to a mono-exponential decay model to calculate ${T1}_{\rho}$ values on a pixel-by-pixel basis [2]. The corresponding mFI maps were then calculated with the use of Eq. (1) on a pixel-by-pixel basis. Segmental [3] ECVvalues, mFI values and ${T1}_{\rho}$ values were all acquired. Whole-heart average values were obtained, with the exclusion of artifact segments. The artifacts exclusion of ${T1}_{\rho}$ is based on original ${T1}_{\rho}$-weighted images. This artifact is the result of B_1_ inhomogeneity. Signal intensity values of more than the mean plus 5 multiplied by the standard deviation of the signal intensity in remote normal myocardium were used to determine regional fibrosis.

**REFERENCES**

1 Zeng W, Wen X, Gong L et al (2015) Establishment and ultrasound characteristics of atherosclerosis in rhesus monkey. Biomed Eng Online 14 Suppl 1:S13

2 Witschey WR, Pilla JJ, Ferrari G et al (2010) Rotating frame spin lattice relaxation in a swine model of chronic, left ventricular myocardial infarction. Magn Reson Med 64:1453-1460

3 Cerqueira MD, Weissman NJ, Dilsizian V et al (2002) Standardized myocardial segmentation and nomenclature for tomographic imaging of the heart. A statement for healthcare professionals from the Cardiac Imaging Committee of the Council on Clinical Cardiology of the American Heart Association. Circulation 105:539-542

| **Supplemental Table 1. Echocardiography Characteristics** | | | | |
| --- | --- | --- | --- | --- |
|  | **Healthy Control Animals**  **(N=9), Group 1** | **Monkeys with Type 2 Diabetes and Mild Diastolic Dysfunction**  **(N=9), Group 2** | **Monkeys with Type 2 Diabetes and Moderate Diastolic Dysfunction**  **(N=9), Group 3** | ***P**** **Value** |
| Heart rate, beats/min | 129.33 ± 21.97 | 117.61 ± 13.67 | 131.95 ± 27.37 | .484 |
| E, cm/s | 79.72 ± 7.89 | 67.12 ± 15.37‡ | 83.27 ± 12.61† | .026 |
| A, cm/s | 57.95 ± 7.66 | 68.26 ± 9.16 | 61.92 ± 11.74 | .095 |
| E/A | 1.40 ± 0.24 | 0.98 ± 0.23‡ | 1.41 ± 0.40† | .008 |
| E’, cm/s | 9.05 ± 1.31 | 8.22 ± 1.30 | 5.62 ± 0.89‡† | .000 |
| A’, cm/s | 5.95 ± 1.26 | 6.49 ± 1.10 | 6.61 ± 1.66 | .548 |
| E/E’ | 8.88 (8.10 - 9.10) | 7.91 (7.68 - 9.17) | 15.28 (12.88 - 18.03) ‡† | .000 |
| E’/A’ | 1.40 (1.25 - 1.78) | 1.20 (1.11 - 1.33) | 0.83 (0.74 - 0.90) ‡ | .001 |
| Values are given as mean ± standard deviation or median (Q1-Q3).  * compared between three groups  ‡ p<0.05 compared with Group 1 (according to the results of Fisher’s Least Significant Difference test and Student-Newman-Keuls test or Dunn-Bonferroni test for post-hoc analysis)  † p<0.05 compared with Group 2 (according to the results of Fisher’s Least Significant Difference test and Student-Newman-Keuls test or Dunn-Bonferroni test for post-hoc analysis)  A, Transmitral late diastolic filling velocity; A’, late diastolic mitral annulus velocity; E, transmitral early diastolic filling velocity; E’, early diastolic mitral annulus velocity; Q, quartile. P-value was the results of One-way analysis of variance (for normally distributed date) or Kruskal-Wallis H test (for non-normally distributed date. | | | | |

| **Supplemental Table 2. Post Hoc Analysis of Basic Characteristics, Echocardiography**  **and Cardiac Magnetic Resonance** | | | |
| --- | --- | --- | --- |
|  | ***P* Value,**  **Group 1 vs. Group 2** | ***P* Value,**  **Group 1 vs. Group 3** | ***P* Value,**  **Group 2 vs. Group 3** |
| FPG, mmol/L | .007 | .000 | .740 |
| HbA1c, % | .001 | .005 | 1 |
| E, cm/s | .041 | .457 | .011 |
| E/A | .007 | .944 | .006 |
| E’, cm/s | .155 | .000 | .000 |
| E’/A’ | .515 | .001 | .057 |
| E/E’ | .855 | .007 | .000 |
| Global T1ρ relaxation time, ms | .018 | .002 | .355 |
| Global mFI | .049 | .000 | .007 |
| Global ECV, % | .307 | .000 | .027 |
| GSrL, 1/s | .203 | .013 | .916 |
| A, Transmitral late diastolic filling velocity; A’, late diastolic mitral annulus velocity; E, transmitral early diastolic filling velocity; E’, early diastolic mitral annulus velocity, ECV, Extracellular volume fraction; FPG, fasting plasma glucose; GSrL, global peak diastolic longitudinal strain rate; HbA1c, glycated hemoglobin; mFI, myocardial fibrosis index. The p value was the result of Fisher’s Least Significant Difference test for normally distributed variables or Dunn-Bonferroni test for non-normally distributed variables. | | | |
